# Essential and non-essential element concentrations in human milk samples and the assessment of infants’ exposure

**DOI:** 10.1038/s41598-024-58683-7

**Published:** 2024-04-07

**Authors:** Agnieszka Bzikowska-Jura, Aleksandra Wesołowska, Piotr Sobieraj, Agnieszka Nawrocka, Aleksandra Filipek, Maciej Durkalec, Danuta Katryńska, Piotr Jedziniak

**Affiliations:** 1 Laboratory of Human Milk and Lactation Research at Regional Human Milk Bank in Holy Family Hospital, Department of Medical Biology, Faculty of Health Sciences, Medical University of Warsaw, Warsaw, Poland; 2 Department of Internal Medicine, Hypertension and Vascular Diseases, Faculty of Medicine, Medical University of Warsaw, Warsaw, Poland; 3 Department of Pharmacology and Toxicology, National Veterinary Research Institute, Pulawy, Poland; 4 The Institute of Biology and Earth Sciences, University of the National Education Commission, Cracow, Poland

**Keywords:** Human milk composition, Breastfeeding, Diet, Lead, Mercury, Cadmium, Metals, Paediatric research, Nutrition

## Abstract

As the data concerning element concentrations in human milk (HM) samples and their intake by infants are lacking in Poland, the present study aimed to explore this issue. The material consisted of HM samples obtained from 30 exclusively breastfeeding mothers during 4–6 weeks postpartum. Additionally, to identify the factors that may potentially affect HM composition, information regarding maternal data (anthropometry, body composition, and diet) was also collected. Maternal diet was assessed with two methods—a food frequency questionnaire and 3-day dietary records. In total, 18 essential and non-essential elements were determined. For the elements analysis, we used inductively coupled plasma quadrupole mass spectrometry. Most of the elements (n = 11, 61%) were detected in all HM samples. In all HM samples tin concentration was higher (5.67 ± 2.39 μg/L) than the usual range reported by the World Health Organization (~ 1.0 μg/L). HM cadmium content was positively associated with maternal salty snacks intake (r = 0.502, p = 0.005), arsenic with whole-grain products intake (r = 0.37, p = 0.043), and mercury concentration with fruits and seeds/nuts consumption (r = 0.424, p = 0.042 and r = 0.378, p = 0.039, respectively). Higher HM lead concentration was predicted by maternal age (95% CI [0.94–0.97]), intake of fish (95% CI [1.01–1.03]), and vegetables (95% CI [1.02–1.06]). The highest infants’ intake was observed for copper (35.24 ± 12.48) and the lowest for arsenic (0.076 ± 0.102). Infants’ exposure to lead was associated with maternal frequency consumption of canned fish (p = 0.0045). There is a need to perform further research on this topic to maximize the benefits of breastfeeding by minimizing maternal and infant exposure to potentially toxic elements.

## Introduction

Human milk (HM) is the best source of nutrition for newborns and infants, as it contains all essential nutrients and additionally, a lot of bioactive components (e.g. antibodies, enzymes, human milk oligosaccharides, hormones) that boost babies’ immune system and protect from many diseases^1,2^. For this reason, the World Health Organization (WHO) recommends exclusive breastfeeding up to 6 months of age and continuing during complementary feeding for at least 2 years^3^. Maternal exposure to different environmental contaminants including heavy metals may lead to their transfer to newborns/infants via HM^4^. The occurrence of chemical contamination in HM may be influenced by dietary habits, place of residence, cigarette smoking, amalgam fillings, and occupational exposure of women^5–7^. Other factors that may have an impact on their HM concentration are maternal health status (e.g. the presence of intestinal parasites), age, and lactation period^8,9^. Considering the changes in maternal homeostasis during lactation, breastfeeding women are more susceptible to side effects from toxic elements including arsenic, cadmium, mercury, and lead^10^. It also applies to infants, mainly due to the immaturity of their gastrointestinal tract, especially the function of liver detoxification^11^. The toxic impact of heavy metals is complex and can affect the central nervous system, liver, kidneys, lungs, and immune system^5^.

Regarding the possible excessive exposure of infants to specific elements, the WHO analyzed 24 elements in HM samples that came from six different countries (Nigeria, Guatemala, Zaire, Philippines, Sweden, and Hungary), and on this basis established the ‘normal condition levels’ for all trace elements^12^. Therefore, the first objective of our study was to analyze selected essential and non-essential elements: aluminum (Al), arsenic (As), barium (Ba), beryllium (Be), cadmium (Cd), chromium (Cr), cobalt (Co), copper (Cu), lead (Pb), manganese (Mn), mercury (Hg), molybdenum (Mo), nickel (Ni), thallium (Tl), thorium (Th), tin (Sn), uranium (U), vanadium (V) in Polish HM samples and compare the results with WHO reference values. Then, we aimed to assess the mutual relationship between essential and non-essential elements, estimate their intake by infants, and analyze the correlations with maternal factors.

## Material and methods

### Study design

We randomly recruited 30 exclusively breastfeeding mothers from the Department of Obstetrics and Gynecology at Holy Family Hospital in Warsaw, Poland. The inclusion criteria involved: age ≥ 18 years, singleton and full-term pregnancy, 4–6 weeks postpartum, no chronic diseases, no smoking during pregnancy and lactation, and no contradiction to body composition analysis (e.g. pacemaker, epilepsy). At enrolment, data on mother and infant demographic characteristics were collected during a face-to-face interview with the researcher (AB-J). Additionally, all women were instructed in milk collection guidelines, and a nutritional questionnaire was conducted. All maternal measurements and data collection were performed within a maximum of 7 days from the recruitment, during maternal visits in the Laboratory of Human Milk and Lactation Research, Medical University of Warsaw. Maternal actual body weight and height were measured using a Seca 799 electronic column scale (± 0.1 kg/cm; Seca, Chino, CA, USA). Body composition analysis was based on bioelectrical bioimpedance using the Maltron BioScan 920-II (Maltron Bioscan, Rayleigh, UK) and the following parameters were estimated: fat mass, fat-free mass, total body water, extracellular water (ECW), intracellular water (ICW), ECW/ICW ratio, body cell mass, muscle mass, minerals and resting metabolic rate (REE). All women signed a written informed consent to participate in the study. The study protocol was approved by the Ethics Committee of the Medical University of Warsaw (AKBE/241/2022). All the authors confirm that the study was performed following relevant guidelines and regulations (Declaration of Helsinki).

### Maternal nutritional data

The assessment of maternal diet was based on two methods: 3-day dietary records (two weekdays and one weekend day) and a semi-structured food frequency questionnaire (FFQ) which aimed to assess the habitual intake of selected food products. FFQ was developed according to the validity guidelines^13^ and was concerned 3 months before the study. The answers options in FFQ were divided into five categories as follows: ‘every day’ (4 points), ‘more than twice a week but not every day’ (3 points), ‘once or twice a week’ (2 points), ‘less than once a week’ (1 point), ‘less often or never’ (0 points). The data from 3-day dietary records were calculated using Dieta 6.0 software (National Food and Nutrition Institute, Warsaw, Poland), which is the Polish reference method, used in nutritional studies.

### Human milk sampling procedure

The women were instructed to wash the nipple and breast area thoroughly with deionized water. All mothers expressed milk four times during the 24 h (6:00–12:00, 12:00–18:00, 18:00–24:00, and 24:00). Additionally, during each session of milk expression, they were asked to collect fore- and hindmilk (about 5–10 mL). Therefore, finally, about 40–80 mL of pooled milk was collected from every mother. During daily milk collection the milk was stored in the refrigerator (− 4 °C) and then immediately frozen, transported to the Laboratory, and then stored at − 80 °C for further analysis.

### Samples preparation

A portion (0.1–0.5 g) of milk samples was weighed and placed into pre-cleaned Teflon vessels (DAK-40, Berghof), then 3.0 mL of HNO_3_ (65%) and 0.5 mL of non-stabilized H_2_O_2_ was added. After pre-digestion in ambient temperature (~ 30 min), the vessels were closed, placed in the microwave digestion system (Speedwave 4, Berghof, Germany), and digested according to the previously optimized heating program (temperature in °C, ramp time in minutes, hold time in minutes, pressure in Ba) as follows: 175, 15, 10, 50; 230, 5, 15, 90; 50, 1, 10, 40. Cooled digests were then transferred to calibrated 10 mL polypropylene flasks and diluted with MilliQ water. For quality control purposes a reagent and control samples were prepared in each batch.

### Analytical methods—instrumentation

The concentrations of As, Cd, Cr, Cu, Mn, Ni, Pb, Tl, and V in HM were determined in the Department of Pharmacology and Toxicology of the National Veterinary Research Institute in Pulawy by the inductively coupled plasma mass spectrometry technique (ICP-MS) using 7700× spectrometer (Agilent Technologies, Tokyo, Japan) equipped with a cyclonic spray chamber, MicroMist nebulizer, and quartz torch. The ASX-520 autosampler (Teledyne CETAC Technologies, Omaha, NE, USA) was used for sample introduction. The quantification of V, Cr, Ni, Cu, As, Cd, Tl, and Pb was based on a five-point external calibration curve (from 0.5 to 250 μg/L) prepared by dilution of multi-element standard stock solution (10 µg/mL) IV-ICPMS-71A (Inorganic Ventures, Christiansburg, VA, USA). The multi-element standard solution containing 200 µg/L of Bi, Li, Ho, In, Rh, Sc, Tb, and Y, prepared by diluting 6020 ISS stock solution (Inorganic Ventures, Christiansburg, VA, USA), was used as an online internal standard. The ICP-MS was optimized daily with a tuning solution for ICP-MS 7500cs (Agilent Technologies, Santa Clara, CA, USA), containing 1 μg/L of Ce, Co and Y. The trueness of the calibration curve was monitored using the Trace Elements in Water certified reference material SRM-1643f (NIST, Gaithersburg, MD, USA). Mercury analysis was performed by atomic absorption spectrometry using a DMA-80 mercury analyzer (Milestone, Soristole, Italy). The quality of measurements was verified by the analysis of following CRMs: ERM-BD150 Skimmed milk powder (low) (IRMM, Geel, Belgium), ERM-BD151 Skimmed milk powder (high) (IRMM, Geel, Belgium), DORM-4 Fish Protein (NRC CNRC, Ontario, Canada), Seronorm Serum L-1 (SERO, Billingstad, Norway), TORT-3 Lobster Hepatopancreas (NRC CNRC, Ontario, Canada). Recoveries of the studied elements were within the range of 70–120%. A Speedwave 4 (Berghof, Eningen, Germany) microwave digestion system equipped with DAP-40 high-pressure TFM™-PTFE vessels (pressure range: 0–40 bar) was used for wet digestion. The Limits of detection (LODs) and limits of quantification (LOQs) of the method used are listed in Table 1. The results are expressed in mg/kg of wet weight (wet wt).

**Table 1:** Limits of detection (LODs) and limits of quantification (LOQs) of tested elements.

Element	LOD (mg/kg)	LOQ (mg/kg)
Al	0.066	0.15
As	0.0012	0.002
Ba	0.0008	0.001
Be	0.0014	0.003
Cd	0.0013	0.002
Co	0.0015	0.002
Cr	0.0011	0.002
Cu	0.0021	0.004
Hg	0.0006	0.001
Mn	0.0018	0.003
Mo	0.010	0.015
Ni	0.0015	0.002
Pb	0.0011	0.002
Sn	0.0007	0.001
Tl	0.0006	0.001
Th	0.0006	0.001
U	0.0007	0.001
V	0.0015	0.002

### The infants’ exposure—daily intake

As suggested by Wooldridge et al.^14^, we asked all mothers to weigh their infants before and after each feeding, with a level accuracy of 0.1 g. Based on these measurements, we calculated the infants’ daily milk intake. Then, we estimated the exposure to essential and non-essential elements. The calculation was based on elements’ concentrations in HM, infants’ weight, and consumed daily milk volume^15^, the equation was as follows:

$${\mathbf{Estimated}} \, {\mathbf{daily}} \, {\mathbf{intake}} \, \left( {{\mathbf{EDI}}} \right)[{\text{mg}}/{\text{kg \,\,body \,\,weight}}/{\text{day}}] \, = \frac{{{{{\rm Milk \,\,volume} * {\rm Element}^{\prime}{\rm s \,\,concentration} }}}}{{{{{\rm Infant}^{\prime}{\rm s\,\, body \,\,weight}}}}}$$

### Statistical analysis

Continuous variables were described using the mean and standard deviation (SD) and as medians with interquartile ranges. In case of elements’ concentrations lower than the limit of detection, the mean, median, and percentiles of analyzed variables were computed with the exclusion of these values. Qualitative variables were presented as numbers and percentages within respective categories. To evaluate whether the data follows a normal distribution the Shapiro–Wilk test was used. Correlation analysis between variables employed Pearson's correlation coefficient or Spearman's rank correlation coefficient depending on the distributional characteristics of the variables.

To evaluate the impact of maternal and dietary factors on analyzed essential and non-essential elements concentration in HM multivariable logistic regression models were built. The predicted variable exceeded the intake norm of a particular element and considered explanatory variables were maternal age, maternal fat percentage, and frequency intake of selected food groups. The methodology involved the systematic development of models and subsequent selection based on the Akaike Information Criterion (AIC) using an exhaustive method (package ‘glmulti’ for R). The computations were performed using R version 4.1.0 (The R Foundation for Statistical Computing, Vienna, Austria).

## Results and discussion

### Characteristics of the population

The median age of the participating women was 32.5 years, all of them had university education and lived in the industrial area. Most of them (n = 20, 67%) had a normal weight before pregnancy (BMI = 18.5–24.9 kg/m^2^), whereas 7 (23%) were overweight or obese (BMI ≥ 25.0 kg/m^2^). The mean infants’ birth weight was 3573.7 ± 445.7 g. The mean infant’s milk intake was 502.1 ± 58.8 mL/day. Detailed data regarding maternal and infant characteristics is presented in Table 2.

**Table 2:** Characteristic of the mothers and infants.

Characteristic	Mean ± SD	Median (interquartile ranges)
Mothers		
Age (years)	33.7 ± 4.8	32.5 (30–36.5)
Pre-pregnancy body weight (kg)	62.8 ± 12	61 (53.9–71.9)
		22.6 (19.8–24.2)
Current body weight (kg)	65.7 ± 13.4	64.8 (54.2–77.5)
Current BMI (kg/m^2^)	23.7 ± 3.8	23.5 (20.4–26.9)
Fat free mas (%)	70.9 ± 9.3	70.3 (63.7–79.8)
Fat mass (%)	29.1 ± 9.3	29.7 (20.2–36.3)
Total body water (L)	51 ± 5.6	50 (45.9–55.5)
Extracellular water—ECW (L)	46.4 ± 3.0	46.7 (45.4–48)
Intracellular water—ICW (L)	53.5 ± 3.0	53.3 (52–54.6)
ECW/ICW	0.9 ± 0.1	0.9 (0.8–0.9)
Body cell mas (kg)	24.2 ± 3.0	24 (22.2–26.9)
Proteins (kg)	8.7 ± 1.4	8.9 (8.2–9.7)
Minerals (kg)	3.7 ± 0.6	3.7 (3.4–4)
Muscle (kg)	19.8 ± 2.0	19.6 (18.6–21.2)
Infants		
Birth weight (g)	3573.7 ± 445.7	3560 (3225–3867.5)
Current weight (g)	4666.7 ± 391.1	4670 (4355–4897.5)
Milk intake—volume (mL)	502.1 ± 58.8	496.5 (470.2–526.5)

SD, standard deviation.

### Maternal dietary intake

Regarding the Polish Nutritional Standards^16^ the estimated average energy requirement for exclusively breastfeeding women is about 2300–2755 kcal/day. In the present study, in most of the women (n = 21, 70%) energy intake was below 2000 kcal and the median energy value was 1798.4 kcal/day (1524.5–2047.1 kcal/day). The main sources of the total energy value of the diet were carbohydrates (51.1 ± 7.1%) and then fats (31.1 ± 6.1%). Almost half of the mothers (n = 14, 47%) declared taking dietary supplements. Their composition was checked, and nutritional value was involved in the calculation of the maternal diet. Only 6 (20%) and 3 (10%) mothers met the recommended daily intake for calcium and zinc, respectively.

### Essential and non-essential elements concentrations in human milk

In total, the concentration of 18 essential and non-essential elements was assessed in HM samples. The following elements: As, Cd, Cr, Cu, Hg, Mn, Mo, Ni, Sn, Pb, and Tl were detected in all tested milk samples (n = 30), whereas concentrations of Al, Ba, Be, Co, Th, U, and V were below the limit of detection of the method. Detailed information about the concentration of analyzed elements and the number and percentage of left-censored data is presented in Table 3. Considering the high proportion of left-censored results of V (90%), the descriptive statistics for this metal were not calculated. Among elements that were detected in all samples, the highest mean concentration was found for Cu (323.7 μg/L) and the lowest for Tl (0.2 μg/L).

**Table 3:** The concentration (μg/L) of trace elements in human milk samples (N = 30).

Element	N < LOQ (%)	Mean ± SD	Q5–Q10	Median (Q25–Q75)	Q90–Q95	Min–Max	% of samples > usual levels^a^
Al	21 (70)	305.3 ± 353.8	7.74–14.0	221.9 (128.9–363.5)	557.3–867.1	0.00–1177.02	No data
As	0	0.7 ± 1.0	0.11–0.15	0.4 (0.2–0.7)	1.18–2.11	0.07–5.31	36.7% (0.2–0.6)
Ba	1 (3)	2.84 ± 1.75	0.50–0.76	2.6 (1.6–3.47)	5.14–5.96	0.00–7.09	No data
Be	6 (20)	0.08 ± 0.09	0.01–0.01	0.05 (0.03–0.103)	0.19–0.21	0.01–0.43	No data
Cd	0	0.2 ± 0.1	0.14–0.16	0.2 (0.2–0.3)	0.34–0.37	0.13–0.41	0% (< 1.0)
Co	9 (30)	0.07 ± 0.08	0.01–0.01	0.05 (0.02–0.08)	0.16–0.19	0.00–0.36	3.3% (0.15–0.35)
Cr	0	1.3 ± 1.0	0.48–0.56	1 (0.7–1.3)	2.20–4.05	0.41–4.12	23.3% (0.8–1.5)
Cu	0	323.7 ± 98.0	180.0–189.0	332.8 (240.4–408.1)	438.2–457.4	118.38–473.47	63.3% (180.0–310.0)
Hg	0	0.5 ± 0.1	0.30–0.30	0.4 (0.4–0.6)	0.60–0.60	0.30–0.80	0% (1.4–1.7)
Mn	0	2.47 ± 1.91	0.55–0.86	1.64 (1.14–3.80)	5.88–6.13	0.07–6.49	23.3% (3.0–4.0)
Mo	0	1.19 ± 1.07	0.23–0.33	0.83 (0.60–1.23)	3.03–3.41	0.18–4.32	13.3% (0.3–3.0)
Ni	0	5.4 ± 3.5	2.05–2.23	3.9 (3.0–7.1)	10.90–12.14	1.54–14.49	0% (11.0–16.0)
Sn	0	5.67 ± 2.39	4.28–4.33	4.985 (4.56–5.66)	6.53–10.24	4.2–15.07	100% (~ 1.0)
Pb	0	4.8 ± 0.8	3.76–3.87	4.6 (4.4–5.2)	5.55–6.03	3.66–6.96	33.3% (2.0–5.0)
Th	20 (67)	0.10 ± 0.21	0.01–0.01	0.02 (0.01–0.06)	0.20–0.44	0.00–0.68	No data
Tl	0	0.2 ± 0.5	0.02–0.03	0.1 (0.1–0.1)	0.25–0.62	0.02–2.60	No data
U	14 (47)	0.03 ± 0.05	0.01–0.01	0.02 (0.01–0.02)	0.04–0.09	0.00–0.23	No data
V	27 (90)	–	–	–	–	–	–

^a^Usual levels (μg/L) are provided in brackets next to percentage values^12^.

The obtained results were compared with the usual reference levels provided by the WHO^12^. In all samples, Sn concentration was above the reference value of 1 μg/L. The review of the recent literature showed that data on Sn levels in HM remains scarce. A multicenter WHO study reported that Sn concentration ranged from 1.4 to 3.3 μg/L^12^. Although our results exceeded these values, even higher concentrations were obtained in the latest Iranian study (7.4 ± 3.77 μg/L)^17^. Additionally, the authors^17^ reported that Sn content was positively correlated with maternal BMI (p = 0.036), in our study, we did not observe such a relationship (r = 0.122, p = 0.519). Comparing the concentration of other elements with WHO levels, no exceedances were found for Cd, Hg, and Ni (Table 3).

Recently Ghane et al.^5^ provided a systemic review and meta-analysis to report the concentration of potentially toxic elements (As, Cd, Cu, Fe, Ni, Pb, Zn) in HM. The authors observed that the highest concentration of As (2800 μg/L) and Pb (268 μg/L) were related to the Western Pacific Region, whereas Cd (70 μg/L) to the European Region. Considering trace elements, the highest contents of Cu (1840 μg/L) and Ni (600 μg/L) were observed in the Eastern Mediterranean Region. What is important, the results of Cochrane’s Q test and I^2^ statistics indicate a significant heterogeneity (p < 0.001 for all metals) among included studies in terms of element concentrations in HM. The lowest and the highest concentrations of metals were related to Cd (150 μg/L) and Cu (1840 μg/L), respectively, which is consistent with our results. However, in our study, the obtained mean concentrations were significantly lower for both metals (0.2 μg/L and 323.7 μg/L, respectively).

Comparing our results with others which come from European countries, we found that the mean concentration of Cd was similar to values reported in Greece (0.19 ± 0.15 μg/L)^18^, lower than those in Spain (0.4 ± 1.6 μg/L)^19^ and previously reported in Poland (2.114 ± 2.112 μg/L)^20^, but higher than in Sweden (0.086 ± 0.045 μg/L)^21^. We did not find any relationship between Cd concentration and maternal factors, including anthropometric data and current dietary intake, however significant positive correlation was reported for habitual salty snacks intake (r = 502, p = 0.0047). The main dietary sources of Cd are plant-based food, mainly potatoes, which are the basis for chips production, which may explain our results^22^. Contrary to these findings, Motas et al.^19^ reported that Cd concentration was negatively associated with maternal age (p = 0.012) and current weight of the baby (p = 0.038) and positively correlated with maternal current or past smoking habits (p = 0.014) and following vegetarian diets (p < 0.01). In turn, Leotsinidis et al.^18^ reported higher Cd content in samples of mothers with high vegetable intake. On the other hand, Nakhaee et al.^17^ and Ursinova and Masanova^23^ did not observe any associations between maternal factors and Cd content in HM.

Considering the As level in HM, the literature data are rather limited. We detected As in all HM samples, contrary to American^24^ and Spanish^19^ studies, in which this metal was detectable in 56% and only 12% of analyzed samples, respectively. One of the reasons for this discrepancy are different LOD for this metal, which was 0.0012 μg/L in our study and 0.22 μg/L in the American study. Motas et al.^19^ did not provide the LOD. Interestingly, the mean concentration of As in our study (0.7 ± 1.0 μg/L) was lower than those reported by Motas et al. (0.9 ± 2.7 μg/L)^19^ who found a large variance in As levels in the tested population. Similarly to our results, a recent Swedish study^21^ showed that As was detected in all tested samples of HM (0.55 ± 0.7 μg/L; LOD = 0.007 μg/L). The main maternal dietary sources of As are drinking water, grain, and grain-based products, mainly rice-based products^25^. In our study, we observed that As content in HM was weakly correlated with maternal whole-grain product consumption (r = 0.370; p = 0.044). No other correlations between As levels in HM and maternal factors were found.

The levels of Pb found in our study (Table 3) were consistent with those reported from Slovakia (4.7 μg/L)^23^, but higher than results obtained in Turkey (2.27 ± 0.36 μg/L)^11^, Portugal (1.55 ± 1.38 μg/L)^26^, and Greece (0.48 ± 0.6 μg/L)^18^. We hypothesized that Pb present in HM originated mainly from maternal bone deposits. During the second trimester of pregnancy, when the fetal skeletal is developing, Pb enters to maternal blood along with calcium mobilization from the bones. Then, Pb is resorbed from maternal bone stores to the bloodstream and HM to compensate for calcium needs during breastfeeding^27^. Performed in our study multivariable logistic regression analysis showed that higher HM lead concentration was predicted by maternal age (95% CI [0.94–0.97]) and intake of fish (95% CI [1.01–1.03]) and vegetables (95% CI [1.02–1.06]). Considering food consumption, other authors found that intake of cereals, fish^28^, red meat, cheese, rice^17^, coffee, and dairy products^29^ affects Pb content in HM. In turn, Nassir et al.^30^ and Cherkani-Hassani et al.^15^ reported that the milk of mothers who consume well water had a lower concentration of Pb in comparison to those who used tap water for drinking.

Sharma et al.^31^ reported a significant decline in Hg concentration in HM between 1966 and 2015. These global findings are consistent with observations of several national monitoring studies, eg. in Sweden^32^, Slovakia^23^ or the Republic of Korea^33^, as well as a recent review concerning biomonitoring of terrestrial environments^34^ and extensive study covering ten years of official monitoring of food of animal origin in Poland^35^. Mercury level was previously reported in only one Polish study^36^ which revealed a significantly higher mean concentration (4.4 μg/L). However, it is worth mentioning that the standard deviation was very high (4.4 ± 9.1 μg/L), which means that the values varied greatly. In Germany^37^, the Hg concentration ranged from < 0.2 to 6.86 μg/L (median 0.37 μg/L), which is similar to our results (mean value was 0.5 ± 0.1 μg/L), whereas in Austria^28^ the mean concentration was higher and mean values ranged from 1.07 to 2.17 μg/L (depending on the region). In Slovakian samples^23^ mean Hg concertation amounted to 0.94 μg/L and the only factor affecting its content was the number of maternal teeth feelings (more or less than 7 tooths), 1.08 vs 0.84 μg/L, respectively (r = 0.26; p < 0.01). The Austrian^28^ and Moroccan^38^ authors reported that instead of the region, the HM Hg concentration was affected by the maternal consumption of cereals and vitamin supplementation during pregnancy, whereas smoking did not influence the Hg content in HM. In our study group, positive correlations with plant-based food intake, such as fruits and nuts/seeds were observed (r = 0.424; p = 0.042 and r = 0.378; p = 0.039, respectively). These results have been also confirmed in multivariable logistic regression analysis—intake of fruits (95% CI [1.02–1.06]) and nuts/seeds (95% CI [1.03–1.12]) affected HM Hg concentration. Similarly, Kalemba-Drozdz^36^ observed higher Hg concentration in Polish milk samples from vegan mothers in comparison to omnivorous women. Our results were interesting, since in general, fruits showed lower metal accumulation compared to vegetables^39^. The reason given to explain this result may be the fact that in our group, maternal fruit intake was higher than consumption of vegetables (FFQ results: 17.4/20 points vs 14.2/20 points, respectively).

In Table 4 correlations between element concentrations in HM and selected maternal factors are presented.

**Table 4:** Correlations between selected elements and maternal factors.

Maternal factors	Elements				
	Cd	As	Pb	Hg	Cr
Age (years)	− 0.002	0.001	− 0.316	− 0.326	− 0.080
Pre-pregnancy BMI (kg/m^2^)	0.044	0.090	− 0.114	0.032	− 0.208
Current BMI (kg/m^2^)	0.035	0.031	− 0.053	0.087	− 0.319
Fat mass (%)	0.066	0.122	− 0.163	0.189	− 0.299
Frequency intake of food prodcuts^a^					
Fish and seafood	− 0.017	− 0.183	0.192	− 0.162	− 0.024
Meat	0.020	− 0.019	− 0.055	− 0.061	− 0.162
Grain products	0.032	0.370*	0.091	0.072	0.326
Fruit	0.136	0.117	0.163	0.441*	0.072
Vegetables	0.116	0.203	0.215	− 0.067	0.335
Nuts and seeds	0.014	0.030	0.115	0.473*	− 0.069
Salty snacks	0.366*	− 0.065	0.066	0.234	0.127

Spearman’s correlations coefficient.

^a^Based on food frequency questionnaire (FFQ).

*p < 0.05.

**Figure 1:**
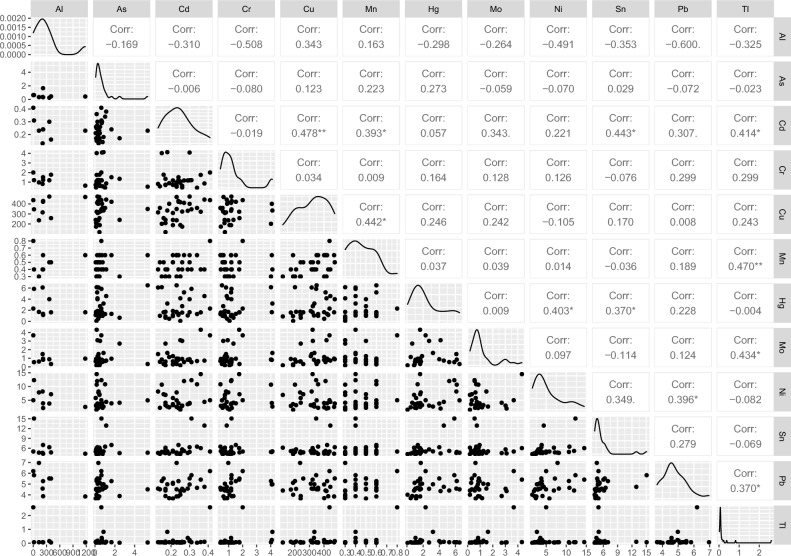
Pairways correlation coefficient of selected elements in analyzed human milk samples.

The correlation between the elements was also examined and V, Cu, Cd, B, and Pb showed normal distribution (Pearson correlation was considered). For the rest elements, considering non-normal distribution, the Spearman correlation was applied. The highest positive correlations were found between Mn and B (r = 0.527; p = 0.0033); Ni and Sn (r = 0.515; p = 0.004); Pb and Sn (r = 0.513; p = 0.004). Pb concentration was also positively related to the content of Ni (r = 0.493; p = 0.0056), Cr (r = 0.468; p = 0.009), and Mn (r = 0.411; p = 0.024). Interestingly, opposite to our results, in the Turkish study^11^ negative correlation between Pb and Cr was found (r = − 0.328; p < 0.05). Additionally, in the present study, Cu concentration was correlated with three other elements Cd (r = 0.478; p = 0.0076), Hg (r = 0.418; p = 0.021), and Be (r = 0.427; p = 0.037). The correlations of 11 essential and non-essential among trace elements in studied HM samples are presented in Fig. 1.

### Infants’ intake of analyzed elements

Based on the elements’ concentration in HM and the volume of milk intake, infants’ exposure to tested elements was calculated (Table 5). The highest mean intake was for Cu (35.24 ± 12.48 μg/kg body weight/day). A similar value was for Al (22.71; 12.39–33.25 μg/kg body weight/day), however, it must be stressed that this element was detected in only 9 samples. Considering elements detected in all samples, the lowest exposure was for As (0.045; 0.025–0.08).

**Table 5:** Heavy metals and trace elements intake by infants** (**μg/kg body weight/day).

Elements	Mean ± SD	Median (interquartile ranges)	Min.–Max.
Al	33.50 ± 43.47	22.71 (12.39–33.25)	0.00–143.82
As	0.076 ± 0.102	0.045 (0.025–0.08)	0.007–0.511
B	0.309 ± 0.193	0.275 (0.161–0.418)	0.00–0.739
Be	0.009 ± 0.012	0.006 (0.003–0.01)	0.00–0.058
Cd	0.026 ± 0.01	0.025 (0.02–0.031)	0.014–0.055
Co	0.008 ± 0.01	0.005 (0.002–0.008)	0.00–0.048
Cr	0.141 ± 0.111	0.105 (0.075–0.158)	0.046–0.522
Cu	35.24 ± 12.48	35.04 (24.12–43.25)	12.06–58.80
Hg	0.309 ± 0.193	0.275 (0.161–0.418)	0.028–0.107
Mn	0.265 ± 0.208	0.178 (0.112–0.351)	0.009–0.712
Mo	0.129 ± 0.12	0.083 (0.062–0.141)	0.018–0.493
Ni	0.582 ± 0.399	0.436 (0.323–0.727)	0.186–1.63
Sn	0.616 ± 0.292	0.548 (0.476–0.626)	0.416–1.819
Pb	0.515 ± 0.099	0.5 (0.45–0.536)	0.351–0.833
Th	0.012 ± 0.028	0.002 (0.001–0.006)	0.00–0.091
Tl	0.023 ± 0.063	0.007 (0.006–0.011)	0.002–0.349
U	0.023 ± 0.063	0.007 (0.006–0.011)	0.00–0.31

Elements intake from human milk was calculated by multiplying their concentration and milk intake (mean value is reported in Table 2).

According to the European Food Safety Authority (EFSA)^40^, the information concerning Cd intake by infants is lacking. Based on the results of only two studies, infants exposure was estimated at the level of 2.61–2.74 μg/kg body weight/week (0.37–0.39 μg/kg body weight/day), which is significantly higher than the results reported in our study. It may result from the fact that in our study we included infants at the age of 4–6 weeks, whereas in the next weeks the volume of consumed milk increases which will translate into higher Cd intake. On the other hand, similar results (mean values: 0.05–0.07, depending on the maternal age) were obtained in the study performed by Shawahna et al.^41^, in which most of the infants were over six months of age.

European data^42^ suggests that Pb intake in infants ranges from 0.21 to 0.94 μg/kg body weight/day, which is similar to our results (0.351–0.833 μg/kg body weight/day). However, in the EFSA document, there is no information about the exposure of exclusively breastfed infants. In Slovakian newborns^23^ (milk samples were obtained on the 4th day postpartum) Pb intake was estimated at the level of 0.36–2.03 μg/kg body weight/day. Additionally, the authors reported that in two individual samples, Pb level exceeded provisionally tolerable weekly intakes (PTWI) (25 μg/kg body weight/week) established by FAO/WHO^43^. Nonetheless, according to EFSA Experts [EFSA, 2010] there is no evidence for a threshold for critical Pb-inducted effects and PTWI of 25 μg/kg body weight/week is no longer appropriate and should not be used. In our study, we found that infants’ exposure to Pb was correlated with maternal frequency consumption of canned fish (r = 0.504, p = 0.0045).

Based on the limited data on the occurrence of methylmercury and inorganic mercury in HM in Europe^44^, the exposure for a three-month exclusively breastfed infant ranged approximately from 0.09 to 0.62 μg/kg body weight/week (0.013–0.09 μg/kg body weight/day) with mean HM consumption and at mean occurrence and from 0.17 to 1.29 μg/kg body weight/week (0.024–0.18 μg/kg body weight/day), respectively. For infants with high milk consumption (about 1200 mL/day), the dietary exposure to methylmercury ranged from 0.14 to 0.94 μg/kg body weight/week (0.02–0.13 μg/kg body weight/day) and inorganic mercury − 0.25 to 1.94 μg/kg body weight/week (0.04–0.28 μg/kg body weight/day). The established tolerable weekly intake (TWI) for methylmercury and inorganic mercury is 1.3 μg/kg body weight and 4 μg/kg body weight, respectively^44^. In our study, we assessed the total Hg intake and it was 0.196–0.749 μg/kg body weight/week (0.028–0.107 μg/kg body weight/day) which means that the mean intake of Pb was below TWI for both forms of Hg.

## Conclusions

To the best of our knowledge, it is the first Polish study in which such a wide spectrum of essential and non-essential elements was analyzed in HM samples. Most of the analyzed elements (61%) were detected in all HM samples. Sn concentration in all samples was higher than the ‘usual’ range reported by the WHO, no exceedances were found for Cd, Hg, and Ni. Considering factors affecting HM element concentrations, in general, we did not find any relationships with maternal anthropometric parameters, body composition, and nutritional habits. The only significant correlations were found for As (positively correlated with whole-grain products), Cd (positively associated with salty snacks intake), and Hg concentrations (positively associated with fruits and seeds/nuts intake). Additionally, higher HM Pb concentration was predicted by maternal age and intake of fish and vegetables. Considering the infant’s intake of analyzed elements, the highest was observed for Cu and the lowest for As. Interestingly, infants’ exposure to Pb was associated with maternal frequency consumption of canned fish. Accordingly, there is a need to perform further research on this topic to maximize the benefits of breastfeeding by minimizing maternal and infant exposure to potentially toxic elements.

## Data Availability

The data that support the findings of the present study are not openly available due to reasons of sensitivity and are available from the corresponding author upon reasonable request.
